# Addressing the Challenge of Defining Valid Proteomic Biomarkers and Classifiers

**DOI:** 10.1186/1471-2105-11-594

**Published:** 2010-12-10

**Authors:** Mohammed Dakna, Keith Harris, Alexandros Kalousis, Sebastien Carpentier, Walter Kolch, Joost P Schanstra, Marion Haubitz, Antonia Vlahou, Harald Mischak, Mark Girolami

**Affiliations:** 1 Mosaiques diagnostics and therapeutics, Hannover, Germany; 2 Water and Environment Research Group, School of Engineering, University of Glasgow, Glasgow, UK; 3 Computer Science Department, University of Geneva, Geneva, Switzerland; 4 Laboratory of Tropical Crop Improvement, Katholieke Universiteit, Leuven, Belgium; 5 The Beatson Institute for Cancer Research and Sir Henry Wellcome Functional Genomics Facility, University of Glasgow, Glasgow, UK; 6 Systems Biology Ireland, Conway Institute, Belfield, Dublin 4, Ireland; 7 Institut National de la Santé et de la Recherche Médicale (INSERM), U858, Toulouse, France; 8 Université Toulouse III Paul-Sabatier, Institut de Médecine Moleculaire de Rangueil, Equipe n° 5, IFR150, Toulouse, France; 9 Department of Nephrology, Hannover Medical School, Hannover, Germany; 10 BHF Glasgow Cardiovascular Research Centre, University of Glasgow, Glasgow, UK; 11 Research Foundation, Academy of Athens, Athens, Greece; 12 Department of Statistical Science, University College London, London, UK

## Abstract

**Background:**

The purpose of this manuscript is to provide, based on an extensive analysis of a proteomic data set, suggestions for proper statistical analysis for the discovery of sets of clinically relevant biomarkers. As tractable example we define the measurable proteomic differences between apparently healthy adult males and females. We choose urine as body-fluid of interest and CE-MS, a thoroughly validated platform technology, allowing for routine analysis of a large number of samples. The second urine of the morning was collected from apparently healthy male and female volunteers (aged 21-40) in the course of the routine medical check-up before recruitment at the Hannover Medical School.

**Results:**

We found that the Wilcoxon-test is best suited for the definition of potential biomarkers. Adjustment for multiple testing is necessary. Sample size estimation can be performed based on a small number of observations via resampling from pilot data. Machine learning algorithms appear ideally suited to generate classifiers. Assessment of any results in an independent test-set is essential.

**Conclusions:**

Valid proteomic biomarkers for diagnosis and prognosis only can be defined by applying proper statistical data mining procedures. In particular, a justification of the sample size should be part of the study design.

## Background

The field of biomarker discovery or clinical proteomics has raised high hopes generated by reports on potential biomarkers, which in many cases subsequently could not be substantiated in validation studies [1,2]. Prominent examples are the findings in [3,4]. This development has resulted in large scepticism from both clinicians and regulatory agencies, which will make the application of valid biomarkers into the arsenal of clinical diagnostics even more of a challenge [5,6]. Further, it is now generally accepted that single biomarkers are unlikely to result in major advancements as the complexity of disease cannot be captured by a single marker; instead, a panel of such biomarkers must be employed [7,8]. However, it is equally evident that such a panel must consist of clearly defined and validated biomarkers in order to provide a well defined signature. This raises the issue of the definition of a valid biomarker. As this is obviously of central importance, we have revisited this issue, not only employing theoretical considerations, but also by using a tractable yet realistic case study. The theoretical considerations in this area apply to the following main challenges:

1 Is the change (frequency or abundance) of a certain molecule observed in a proteomics study of disease, the result of the disease, or does it merely reflect an artefact due to technical variability in the pre-analytical steps or in the analysis, biological variability, or bias introduced in the study (e.g. due to lifestyle, age, and gender)?

2 How should we estimate the number of samples required for the definition of likely valid biomarkers?

3 Which algorithms can be employed to combine biomarkers into a multi-marker classifier, and how can the validity of a multi-marker classifier be assessed? Is validation in an independent test set necessary?

In an effort to investigate these issues and propose answers to these questions, we have employed different analysis and statistical strategies towards biomarker definition and validation using a set of data obtained from real samples. While technical differences do exist between proteomics and peptidomics, these approaches investigate a highly similar chemical entity, and the problems and challenges associated with the identification of potential proteomic and peptidomics biomarkers (features significantly associated with the studied physiological or pathophysiological condition) are essentially identical. Therefore, we feel it is appropriate not to distinguish between peptidomics and proteomics throughout this manuscript. Several platforms for proteomics or peptidomics are currently being used in biomarker discovery studies (reviewed in e.g. [9].

We have chosen data from CE-MS as one representative example, due to the following reasons: a) CE-MS is being used in clinical trials and data from CE-MS are applied in clinical decision-making, b) sufficient datasets of CE-MS were available to us, and c) the analytical performance characteristics of the CE-MS platform are well documented [10,11]

In order to permit a rigorous and realistic assessment of the methodology, the study must (i) represent a real proteomic dataset that is acquired using the same technologies and experimental design as for a biomarker study; (ii) be a classification problem with "typical" complexity, but simple enough to be tractable by standard methods; and (iii) permit the deployment of commonly used statistical analysis strategies in order to benchmark them against an unequivocal outcome. Based on these considerations we choose as an example the definition of proteomic differences between apparently healthy adult males and females. This avoids any bias due to a non-verifiable physiological condition in the subjects, since gender can be assessed with close to 100% confidence [12]. This design avoids an important problem in biomarker discovery pipelines: the so called verification bias. This bias occurs if subjects are not equally likely to have the diagnosis verified by a gold-standard test and if selection for further evaluation is dependent on the diagnostic test result. Of course, in general the clinical situation will not allow for such a sharp definition as in the male-female case, but standard methods exist for accounting for the verification bias if the clinical readout cannot be assigned with 100% confidence [13-15]. We also used a cohort of subjects with diabetes type II either with normal kidney function (controls, CD) or diabetic nephropathy (cases, DN) to demonstrate the applicability of the methods to a case where the clinical readout may not be verified with 100% confidence. The difference in the male-female study turned out to be more subtle than in the CD versus DN case, as the differences between the proteomic profiles between males and females are less pronounced than in the CD-DN case.

As body fluid to be analysed we have chosen urine. The urinary proteome/peptidome is of high stability, reducing pre-analytical variability [16]. CE-MS was chosen as technology as it allows for the routine analysis of a large number of samples, and has been thoroughly validated as a platform technology for proteomic biomarker studies [17]. As result of the current study we demonstrate the importance of a strict and correct use of statistics, especially adjustment for multiple testing. We further describe algorithms that enable prediction of the number of samples required for the definition of biomarkers with high confidence. The results presented here also show that different machine learning algorithms perform similarly (and very well) in establishing discriminatory multi-marker models. However, it is equally evident that these only lead to meaningful results if the number of data points employed is sufficient to learn the difference between the groups, and that the performance of such models can only be assessed on an independent test set. Although our results have been obtained with a particular proteomic technology, CE-MS, the principal conceptual considerations, and hence also the conclusions, are independent of the technology used. Therefore, the results reported here should also be applicable to other datasets generated using alternative standard proteomics technologies such as LC-MS or MALDI. Unfortunately, to the best of our knowledge, there is currently no similar dataset publicly available for MALDI or for LC-MS. Hence, we cannot report on the application of the proposed methods for either platform.

## Results and Discussion

### Biomarker selection

**Figure 1:**
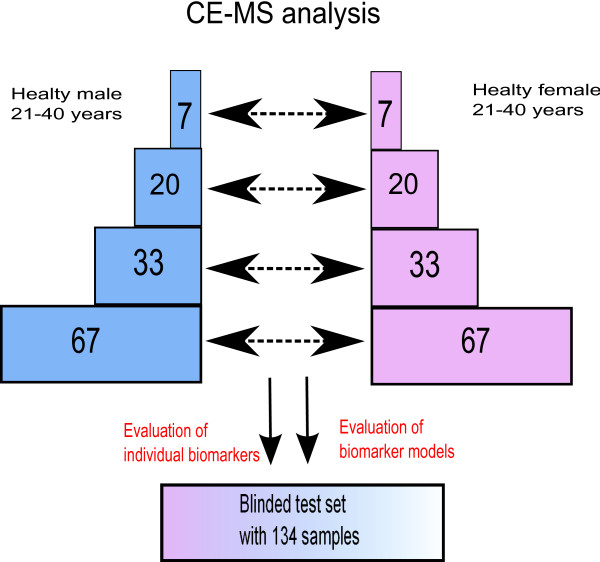
**Study design**. Usage of samples and flow of information. 67 samples from males and females were each employed in a training set for the definition of biomarkers, and establishment of classifiers. Subpopulations of 7, 20, and 33 samples were employed, where indicated. The results (potential biomarkers, classifiers) were evaluated on an independent set of blinded data that also consisted of 67 male and 67 female samples, to enable the best possible assessment.

**Figure 2:**
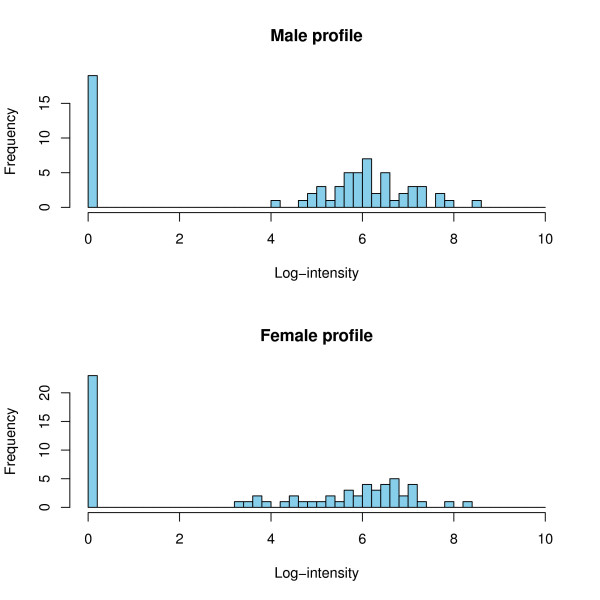
**Typical male and female peptide profiles**. Distribution of a peptide included in the male-female comparative study. The frequency of the peptide ID:4356 is plotted against the natural logarithm of the measured intensity. Both profiles show a point mass at zero and a continuous component. The zero component arises because the peptide is either absent or its concentration is below the detection limit.

**Figure 3:**
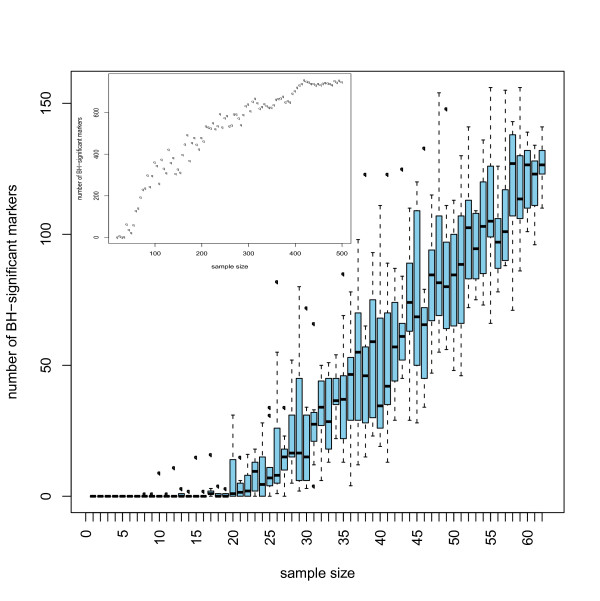
**Number of significant markers depends on sample size**. From the 2 × 67 training data, data sets of sizes *N*_diff _ranging from 7 to 67 were built via resampling. At each sample size the number of significant biomarkers (defined as having a *p*-value after BH adjustments < 0.05) is shown on the vertical axis. The procedure was repeated 10 times to generate the Box-Whisker plots. In all 10 experiments, no biomarkers could be declared significantly differentially expressed below a sample size of 13. On the top left, populations of sizes up to 480 were generated using resampling with replacement, based on the 2 × 67 samples. The figure shows that with sample sizes around 2 × 400 a plateau is reached.

The design of the study is depicted in Figure 1. To detect possible biomarkers, we employed samples from 67 males and 67 females, aged 21-40, as the training set. All relevant data on all samples used in the study are available in the Additional file 1 and Additional file 2. We accepted only peptides that were present in at least 30% of the male or female samples, as a feature with a smaller frequency in both groups may hardly be seen as significantly associated with gender in this study. This threshold resulted in a total of 1216 peptides for further consideration. The appropriateness of a statistical test is primarily determined by the data distribution. Usually, after low-level data processing the resulting data exhibit a mixture distribution characterized by a proportion of observations in a point-mass at zero representing the samples where a peptide is not detected, and a continuous component (see Figure 2). The origin of the point-mass at zero may either be biological, as the protein is really absent in these samples, or technical, as the protein is present but its signal is below the limit of detection (LOD) [18]. The only known fact about the point mass at zero is that those values are between zero and the LOD. In statistical terminology, the proteomics data are left censored. Therefore, usage of standard statistical methods which focus on one part of this mixture at a time can fail to detect differences between classes. The data employed here contains 1169 consonant differences (the group with the higher proportion of zeros has the smaller mean in the continuous component), 38 dissonant differences (the group with the higher proportion of zeros has a larger mean in the continuous component) and 9 without point-mass component. The higher number of consonant markers reflects the fact that markers showing a higher mean are better detectable than those with a mean near to the LOD. A difference in means between the two groups may have its origin in a difference in the proportion of zeros, a difference in the mean of the continuous component, or both. The standard parametric *t*-test may be inappropriate for such data as the underlying assumptions of the test are strongly violated. Non-parametric tests like the Wilcoxon rank sum test (WT) may be more appropriate [19], but may still fail to distinguish the contributions of the two mixture components to the male and female profiles [20]. This suggests the usage of hypothesis tests specifically developed for point-mass mixture data, like the two-part *t*-test, two-part WT and empirical likelihood ratio test, which tests the null hypothesis of no difference in the point-mass proportions and no difference in the means of the continuous components [20]. As expected, owing to differences in statistical power, the number of biomarkers declared statistically significant strongly varies with the type of test adopted (Table 1). When subsequently validating in the hold-out set, the majority of the initially defined potential biomarkers could not be confirmed. This result is likely due to the inherent multiplicity of the problem, strongly supporting the requirement for adjustment for multiple testing [21-23]. These results are even more pronounced when a smaller cohort is employed, resulting in ≤ 10% of the potential biomarkers being confirmed in the test set (data not shown). To control the false discovery rate (FDR) as correction to multiple testing, the Benjamini-Hochberg (BH) procedure was used [24]. In Table 1 we report the number of potential markers with adjusted *p*-values less than 0.05. After adjustment for multiple testing, the WT reports the largest number of significant markers (Table 1). Moreover, 78% of the 112 markers declared significant by the two-part WT are also significant when using the standard WT, indicating that using just standard WT, which is part of standard statistical software (e.g., SAS or SPSS), should enable definition of reliable biomarkers. The fact that many of the values in the profiles are tied to zero only makes the WT conservative and the *p*-values more trustable [25]; as in a pilot study, a false negative is less harmful than a false positive. To test the stability of the significant markers chosen by the different tests, we investigated which of the differentially expressed markers established will still be a valid marker when tested alone on an independent test set (2 × 67 samples). As seen from Table 1 the standard WT has the most markers holding up in the independent test set. Furthermore, the concordance between the biomarkers found in the training set and in the test set is only slightly lower than that for the two-part WT. The results given above argue in favour of using the standard WT for any similar proteomics data. Previous reports have already stressed that non-parametric statistical tests such as the WT may be more appropriate for proteomics data. However, the use of the standard *t*-test is still frequently used and reported in the literature [26-28]. We subsequently investigated the number of potential biomarkers that can be defined when employing only a subset of the original samples. Statistically, if a real difference exists, it may always be detected when the sample size is ad-equate. Hence, studies on small cohorts may over-look important markers. With appropriate sample size all the differentially expressed markers should be detected. Of course, not every difference found with larger sample sizes will be of clinical relevance, hence the need for the incorporation of biological back-ground information. Interestingly, even a subset of the markers found using moderate sample size may still be enough for building a good classifier. As expected, the number of significant markers increases with increasing sample size (see Figure 3). Our simulations, where populations of sizes up to 2 × 480 were generated using resampling with replacement from the 2 × 67 samples, showed that this behaviour stops at sample sizes around 2 × 400 where a plateau is reached (Figure 3 on top left). The concordance of these potential biomarkers in the test set also increases with the sample size. With sample sizes less than 13, no differentially expressed markers are detected at all.

**Table 1:** The number of significant markers depends on the statistical test used

*p*-values	Test	No point-mass	Dissonant	Consonant	Total	Validated	% Validated
unadjusted	*t*-test	3	0	245	248	63	25%
	Wilcoxon	4	5	314	323	109	33%
	Two-part-*t*	3	8	229	240	68	28%
	Two-part-W	4	11	286	301	104	34%
	Empirical LRT	4	7	271	282	81	28%
							

BH-adjusted	*t*-test	0	0	57	57	27	47.3%
	Wilcoxon	3	1	137	141	58	41.1%
	Two-part-*t*	0	3	66	69	30	43.4%
	Two-part-W	3	6	103	112	55	49.1%
	Empirical LRT	2	5	109	116	43	37.0%

The number of potential significant biomarkers when comparing the 67 cases and controls in the training set, based on unadjusted *p*-values (< 0.05) is shown for the *t*-test, WT, Two-part *t*-test, Two-part WT and empirical likelihood ratio test. In addition, the number of consonant, dissonant and no point-mass features among these is listed. All markers defined in the training set were investigated, aiming at validation, in an independent 2 × 67 test set. As is evident, the vast majority of potential biomarkers could not be validated. Lower panel: The number of potential significant biomarkers (*p*-values < 0.05) after BH adjustments for the *t*-test, WT, Two-part *t*-test, Two-part WT and empirical likelihood ratio test, and their performance in the independent test set are shown. While the expectation, that 95% of the potential biomarkers remain significant in the test set, could not be met, the percentage of biomarkers that could be validated is almost 2-fold in comparison to the unadjusted testing.

### Resampling as means to define "better biomarkers"

Variable selection may be seen as the first part in finding a good classifier and must be performed based on training data only. Usually, variable selection is performed only once using all the available training data. This may, however, introduce a substantial bias in declaring a biomarker differentially expressed. This fact is due to the biological variability in the compared populations (here male and female). Cross-validation and Monte Carlo cross-validation (random splitting into learning and test sets) may be adopted to protect the analysis against such a bias. However, as the number of biomarkers may be quite high, these procedures are computationally challenging. Holding out 30% randomly from the 134 male and female training samples and examining the distribution of these biomarkers in the 134 independent test samples, we can detect a clear advantage of the biomarkers that were found with higher frequency in the resamples. From Table 2 we see, provided enough resamplings are done (i.e., *N *≥30), that if a biomarker is found significant in more than 75% of the independent resamples then the chance that it could be confirmed in the test set was between 70 and 100%. However, this procedure also results in a further reduction of the available biomarkers, and appears to be only useful when a rather large number of potential biomarkers should be reduced. In depth analysis of the data indicated that for building classifiers (see also below), a reduction via resampling of the number of biomarkers may not be necessary (data not shown). However, the implementation of such resampling is clearly advantageous for e.g. describing any association with (patho) physiology, as this procedure allows for identifying those biomarkers that show the highest likelihood of actually being associated with the investigated (patho)physiology.

**Table 2:** The concordance of the markers

	All data	30% data hold out						
**# resamples**	**N = 0**	**N = 2**	**N = 10**	**N = 20**	**N = 30**	**N = 40**	**N = 50**	**N = 100**

f(100%)	141	44	16	12	7	8	7	6
f(80%)	141	44	32	27	22	16	20	21
f(50%)	141	125	53	57	49	45	46	47

Concordance in test set								

	58 (41%)	19 (43%)	11 (68%)	10 (83%)	7 (100%)	7 (87%)	6 (85%)	6 (100%)

	58 (41%)	19 (43%)	21 (65%)	18 (66%)	16 (72%)	13 (81%)	15 (75%)	16 (76%)

	58 (41%)	43 (34%)	26 (49%)	30 (52%)	26 (53%)	26 (57%)	25 (54%)	25 (53%)

Concordance of markers detected during resampling with those found significant in the test set: A total of 100 resamples are drawn. For each of the resamples, 30% of the 2 × 67 training samples were held out. The number of significant markers with a *p*-value less than 0.05 after BH adjusted WT test for 50%, 80% and 100% of the resamples is shown. Their concordance in an independent test set of 2 × 67 is also reported in the lower panel. For more than 40 resamples the concordance of the biomarkers remains almost unchanged.

#### Estimation of the sample sizes

An important question in the design of clinical proteomics studies is the selection of an appropriate sample size [29]. The number of units to be included in the study should typically address two issues. First, the differential sample size *N*_diff _should allow the identification of putative biomarkers that are differentially expressed between two conditions (e.g. disease versus control). Second, the discriminative sample size *N*_disc _of the training data should allow the learning of a confident rule for classifying blinded items.

### Estimation of the differential sample size

Here the question is: what is the minimum sample size required to attain a desired statistical power for detecting a meaningful difference between samples? This can be answered by estimation of the differential sample size *N*_diff_. This sample size depends on the false positive rate *α*, typically set at 0.05, the statistical power 1-*β *(e.g. 0.9) and the standardized effect size (e.g. Cohen's *δ *) for quantifying how the classes differ. Indeed, the effect size and its variation turn out to be the most important factors influencing *N*_diff _estimation. Both, effect size and its variation, are traditionally estimated from previously reported experimental data. Unfortunately, in proteomics typically no previous data are available and anticipation of the expected as in [30,31] may hardly be justified. We therefore investigated a resampling-based approach by directly sampling from the pilot data at hand. To simulate a typical proteomics study, we randomly choose 7 samples of each gender from the total training set of 67 males and 67 females. From the 2 × 7 data, we used the bootstrap [32] to generate 2000 sample sets of different larger sizes (2 × 10 to 2 × 120) without any assumption about the underlying distribution of the sampled population. To take into account the multivariate aspect of the problem, we ask for the sample size required for declaring all the markers significant while controlling the FDR at 0.05 using the BH procedure. This is equivalent to conducting the single tests at a more stringent "average type one error" *α*_ave_. Using the result [24]

$$\alpha_{\text{ave}}={\left(1-\beta \right)}_{\text{ave}}q{\left[1+(1-q)\frac{\pi_{0}}{1-\pi_{0}}\right]}^{-1},$$

where (1 - *β*)_ave _is the average power for a single marker (set e.g. to 0.9), *q *is the expected value of the false discovery rate, i.e. *q *= E(FDR), and *π*_0 _is the proportion of markers that are differentially non expressed (true null). To estimate *π*_0 _we use the method described in [33] and fit the observed distribution of the Wilcoxon *p*-values to the following two component model

$$f(p)=\pi_{0}f_{0}(p)+(1-\pi_{0})f_{A}(p),$$

with *f*_0_(*p*) being the density of the null features (that is the differentially non expressed markers) is given by the uniform distribution *U*(0, 1), whereas *f_A_*(*p*) is the alternative density for the differentially expressed markers. Hence we may write

$$f(p)=\pi_{0}+(1-\pi_{0})f_{A}(p).$$

This resulted in a estimate *π*_0 _= 0.5652831 which plugged into Equation 1 leads to *α*_ave _= 0.00223.

**Figure 4:**
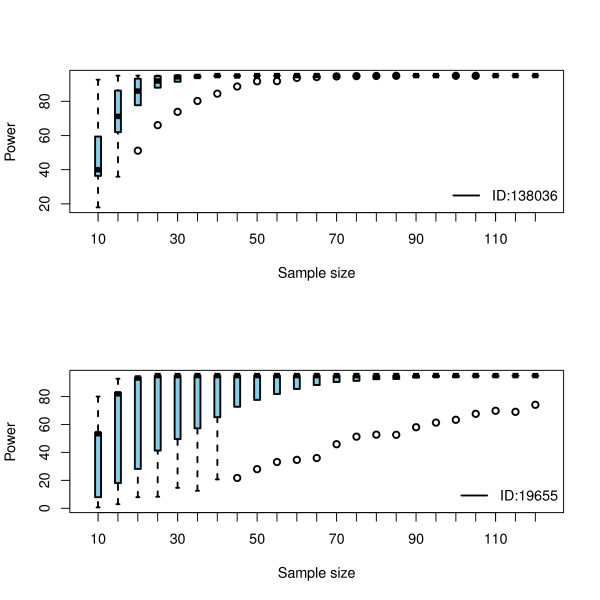
**The resulting power for two markers showing significance after BH adjustments**. The power is calculated as the percentage of times the null hypothesis is rejected. To reach 90% power, *N*_diff _= 30 samples per group is required for ID:138036 (top), whereas for ID:19655 (bottom) *N*_diff _= 15 may be enough. From the original 134 samples, 30 cohorts of 2 × 7 subjects each were randomly built. From each cohort, 2000 resamples of increasing sample size (10-120) were generated via bootstrap with replacement. Circles indicate outliers.

To estimate *N*_diff _we set the value of *α*_ave _= 0.00223 to control the FDR at 0.05 and examined for biomarkers that can be declared significantly differentially distributed. WT was applied to each data set generated. Let N be the number of times the null hypothesis is rejected. Discarding 5% of N (the false positives) is essentially a power estimate. By examining the graphs as in Figure 4 the sample size required for any predefined power can be deduced. Obviously, the more precise the information about the effect size *δ*, the better the trial can be designed. If the sample size is "sufficiently large", then the central limit theorem guarantees that *δ *will be approximately normally distributed. The bootstrap provides a powerful tool to estimate the required differential sample size by directly sampling from the available data and has been shown to give an unbiased estimate of power [34]. However, the key issue here is that the available data be reliable and representative. In the absence of a reliable data set, bootstrapping is not appropriate [35].

In the above considerations, we opted for simplicity for the standard definition of the sample size as the minimum number of samples necessary to achieve a specified power. Alternatively, the "confidence probability formulation" [36] may also be used as it relies on the permutation of pilot study data of small sample sizes.

### Estimation of the discriminative sample size

**Figure 5:**
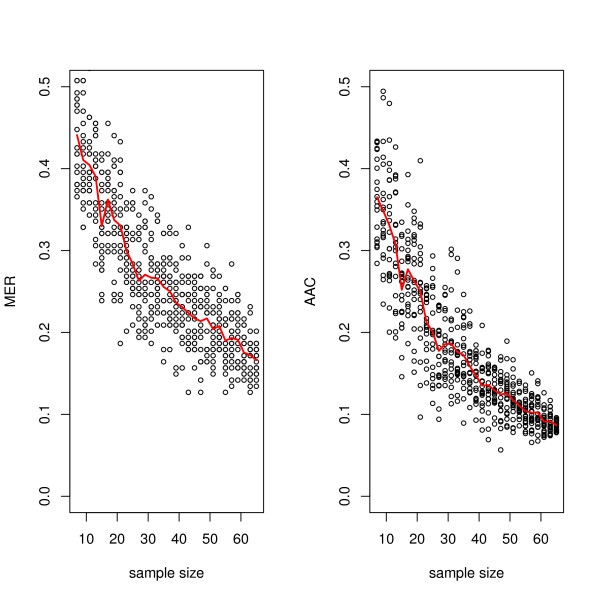
**Learning curve estimation of *N*_disc_**. Cohorts with sample sizes ranging from 2 × 7 - 2 × 65 (given on the x-axis) were arbitrarily generated out of the entire 2 × 67 dataset. 20 repetitions were performed for each size cohort. An SVM-based classifier was built for each dataset and its performance was tested on the independent test set. In the left panel the area above the curve AAC (AAC = 1-AUC) for each classifier is shown. The misclassification error rate MER is shown on the right. The red curves represent the mean AAC and mean MER. The inverse power law behaviour is obvious.

To estimate the effect of training sample size on a classifiers performance we employed learning curves [37]. We used the inverse power-law model

$$E(Y_{\text{train})}=\Gamma +\beta \times N_{\text{train}}^{-\gamma },\;\Gamma ,\beta ,\gamma \geq 0,$$

where *E*(*Y*_train_) is the expected value of a performance metric, e.g. the misclassification error rate MER (MER = 1-ACC, with ACC being the overall model accuracy) or the area above the curve AAC (AAC = 1-AUC), given training sample size *N*_train_. Γ is the minimum classification error that can be expected as *N*_train _→ ∞, the so called Bayes error which provides the lowest achievable error rate for a given pattern classification problem (Γ =Γ(∞)), γ is referred to as the learning rate, and *β *the scale. Using SVM classification, the learning curves for AAC and MER are given in Figure 5. SVM was chosen since this approach has been found to give the best or the near best performance for many microarray data sets [38]. For the actual male and female data, the fit resulted in the equations: $\text{AAC}=0.03+1.39\times N_{\text{train}}^{-0.716}$ and $\text{MER}=0.02+1.052\times N_{\text{train}}^{-0.597}$. From these equations the required sample size can easily be deduced. E.g. for reaching AAC or MER of 10%, *N*_disc _= 65 and *N*_disc _= 75, respectively. Hence, MER seems to overestimate the sample size *N*_disc_, as this quantity holds only for a given threshold whereas the AAC gives a global measure for all thresholds. It is important to note that different classifiers will result in different estimates for the AAC and MER and hence another estimate of *N*_disc _will be obtained.

In practice it is impossible to reach Γ and only upper bound estimates to it can be reached. The aim is to find the discriminative sample size *N*_disc_, that guarantees that Γ (*N*_disc_) of the classifier is within some threshold (e.g ϵ = 5%) from the optimal Bayes classifier obtained for infinite *N*_disc _[39] (that is, Γ(∞) - Γ(*N*_disc_) ≤ ϵ). *N*_disc _may then be obtained by resolving the equation Γ(∞) - Γ(*N*_disc_) = ϵ. Interestingly, here again the effect size *δ *turns out to be the parameter that determines *N*_disc_. In the classification context, the effect size measures the distance between the classes. If the pilot study shows a small effect size then it is unlikely that a good discriminator will be easily obtained. The required *N*_disc _that maximizes the Γ(*N*_disc_) implicitly depends on the false positive rate *α *[39,40]. Consequently, using those markers that control the FDR should generally produce a good classifier [39,40]. For the 67 male and 67 female profiles, controlling the FDR at 0.05 we are able to define 78 significant peptide markers requiring an *N*_diff _< 67. With their calculated effect sizes we found that *N*_disc _= 48 is required to obtain a classifier with 10% performance short of the optimal Bayes classifier. The analytical method described in [39,40] relies on strong distributional assumptions and seems to be less conservative than the learning curve estimation of *N*_disc_.

### Classification

**Figure 6:**
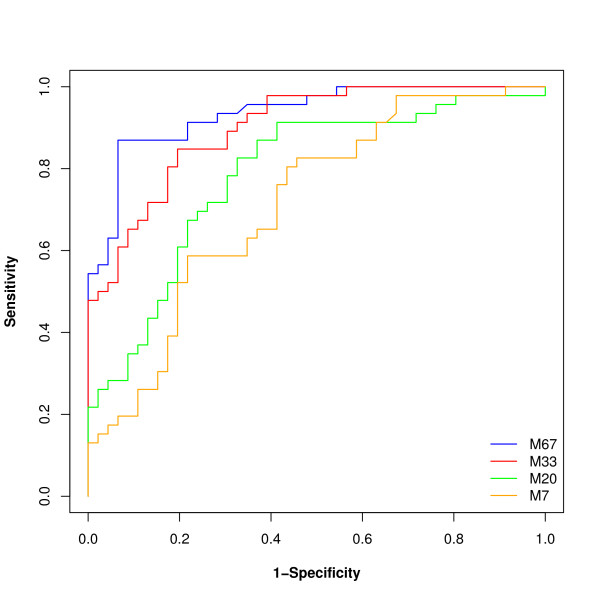
**Classification results of an SVM-based classifier**. Male and female datasets of size 7, 20, 33, and 67 each were compared. Features (selected based on a *p*-value < 0.05 in the unadjusted WT) were combined into respective biomarker models (M7, M20, M33, and M67). Their performance was initially assessed by complete leave-one-out cross validation leading to an accuracy of 100%, 95%, 84% and 94%, respectively, erroneously indicating optimal performance of the M7 model. The ROC analysis shows the results when these models are tested on an independent set of 134 samples. As is evident and expected, best performance can be observed when employing the M67 model, while the M7 model barely exceeds the results obtained by mere guessing (The area under the curve AUC for the models M7, M20, M33 and M67 is 0.715, 0.786, 0.900 and 0.937, respectively).

Once a classification rule has been built, its performance must be evaluated. Frequently, complete leave-one-out cross validation (or similar approaches that all are a reflection of the classifier onto the training set) is employed for error estimation. We have investigated if such an approach is indeed appropriate. An SVM-based classifier was built, based on randomly selected 2 × 7, 2 × 20, 2 × 33 datasets, and the entire 2 × 67 cases and controls. As shown in Figure 6 assessment of the performance based on the complete leave-one-out cross validation (LOOCV) resulted in apparently excellent performance, with the classifier based on 7 cases and controls only appearing to be 100% correct. However, when the classifiers are then tested in the blinded dataset, the results of the classifiers that were built only on a small set of samples could not be verified. As expected, best performance was observed for the classifier based on all available data, where the results from the cross validation and the assessment in the independent dataset are quite similar. These data indicate that results based on the training set only remain questionable, evaluation in an independent set is indeed essential and the ultimate test any procedure must pass. This conclusion supports the findings of [41,42] where the LOOCV error estimate was found to be biased for small samples sizes. For large sample sizes the LOOCV error estimates may be seen as reliable. Therefore we employed the independent test set consisting of 67 male and 67 female samples for evaluation of the performance of all classifiers. The classification results are reported in Table 3. The results suggest that the performance of many machine learning algorithms is quite similar and outperforms a simple tree model. Table 3 also suggests that the use of a generalized linear model (GLM) may not be suitable for similar data. GLM, and the tree model seem to be the more sensitive to the variability and the censored structure of the data.

**Table 3:** Test errors for different classifiers

*N*_train_	SVM	Hierarchical Bayes	AdaBoost	Random Forest	Tree	GLM
7	44.7%	32.8%	43.2%	40.2%	48.1%	41.1%

20	40.2%	30.6%	40.2%	35.8%	43.2%	42.5%

33	32.0%	26.1%	23.1%	34.3%	38.1%	38.5%

67	16.4%	21.6%	14.1%	17.1%	26.0%	46.7%

The test errors for different classifiers and different sizes of the training set (*N*_train_). The results are based on a test set of 67 males and 67 females.

### Applications to the CD-DN case study

To further test the applicability of the reported methods we investigated the difference between CD and DN patients using a data set of 120 CD and 120 DN subjects randomly split into 2 60 training and 2 × 60 test datasets (data available as Additional file 3 and Additional file 4). The differences in this dataset are much more pronounced than the male-female case (Additional file 5). Using the 2 × 60 training data and 10 different random splits we found that on average 447 peptides may be declared differentially expressed using the adjusted WT. 65% of those markers could be validated in the test data (Additional file 6). The fact that using a pilot study of larger size results in more markers being declared significant clearly applies here too, as readily seen from the figure in the Additional file 7. The learning curve of this dataset also shows clearly the inverse power law behaviour (Figure in Additional file 8) and suggests that for the CD-DN case fewer subjects than in the male-female comparison may be required to obtain a classification of comparable performance.

## Conclusions

**Figure 7:**
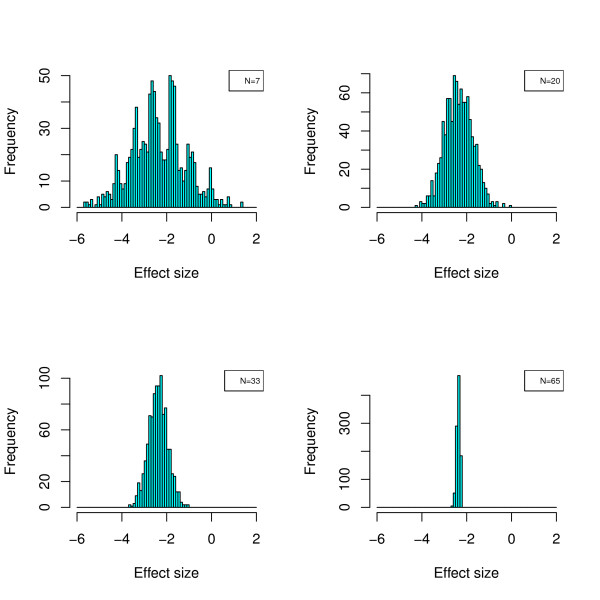
**Effect of sample size on the determination of the distribution of standardized mean-differences**. The distribution of a single peptide, ID:19655, was investigated for four different study designs (N = 7, 20, 33, 67) in 1000 re-sampled distributions out of the complete set of 134 cases and controls each. Typical effect size *δ *= (*μ*_male _- *μ*_female_)/*σ *(with *μ*_male _and *μ*_female_being the mean logarithmic intensity for a given peptide in the male and female population, and the pooled standard deviation) is shown. As is evident, studies of small sample sizes (e.g. N = 7) may determine the log change to be e.g. -5 or even +1, instead of the true change, which is about -2. The consequence of such an error, is that the data obtained from this small training set completely miss the correct effect size and cannot be generalized with even decent confidence.

In this report we have examined what requirements have to be met in order to identify significant proteomic biomarkers and establish classifiers that have a high probability of being valid and can be generalized. To avoid misinterpretation: we did not aim at actually identifying biomarkers that we claim to be gender-associated. The aim of this study was purely to analyse and delineate approaches which ensure a robust study design. In addition, we realize that a study aiming at the identification of biomarkers for classifiers is associated with further challenges, like the above mentioned verification bias. However, some of the main challenges in biomarker discovery may best be investigated using a well defined experimental system, as the one chosen here. In regard to the first major challenge: how to improve the detection of biomarkers clearly associated to disease, we show that the WT test seems to be best suited for this challenge. However, it also is evident that statistical analysis must be adjusted for multiple testing [43], and we demonstrate the deleterious effects of the avoidance of multiple testing. This effect is even more pronounced when only a small number of samples is being used for the analysis. The un-adjusted *p*-values obtained from a small sample set are essentially meaningless, and are not at all connected with the probability of a certain molecule to be a true biomarker in the test set. In fact, the commonly made silent assumption that among the apparently significant biomarkers (based on unadjusted testing), true significant biomarkers can be found with higher probability than in the apparently non-significant group, could not be verified (data not shown). In our dataset the actual significant features were evenly distributed in these two artificial groups (unadjusted *p*-values below and above 0.05), which are only generated due to inappropriate statistics, hence they should be considered to be artefacts. This again underlines the notion that unadjusted *p*-values should not be reported in the absence of other evidence. The lack of statistical power, as well as the unadjusted *p*-values that erroneously are often considered significant, are mostly a consequence of an incorrect estimation of the true distribution. Due to the relatively high variability observed (in the datasets employed here mostly due to biological variability), the true mean cannot be correctly assessed based on a small set of samples. The incorrect distribution suggests significant differences, which in fact are not true. Only upon investigation of a sufficiently large number of samples can the true mean in the cases and controls be determined. This is also evident from the example shown in Figure 7. We also show that confidence in the identified biomarkers can be further improved by resampling of the data, thereby generating a larger number of experiments. Biomarkers that appear significant in each of these experiments, are likely also significant in an independent test set, hence can be generalized. While such a strategy clearly comes at a cost: the number of biomarkers identified is significantly lower, this strategy may nevertheless represent a preferred option to define likely valid biomarkers, due to the high level of confidence that can be reached. Based on a representative proteomic data set, we also presented methods for answering the second important question: how to estimate the required sample size, both for class comparison (differential sample size) and subject classification (discriminative sample size). Our data demonstrate that estimation of the differential sample size required for achieving significance in detecting a certain number of specific biomarkers is possible based on resampling from a relatively small dataset. While we have successfully employed only 7 cases and controls, it seems advisable to slightly increase this number to 12 [44]. A similar strategy may be adopted for estimating the discriminative sample size required for achieving a predefined confidence of a given classifier. Based on the data subsequently obtained, we used the approach of fitting learning curves. This approach may result in an overestimation of the required discriminative sample size. This is in fact beneficial, as it will generally avoid the initiation of an underpowered study. Our data also indicate that testing of biomarkers (e.g. by assessing the *p*-value) or biomarker models (e.g. by cross validation) in the training set will likely result in an overestimation of their quality. As a consequence, it appears that the quality of biomarkers or combinations thereof can only be addressed with confidence in an independent test set. Even when analysing a significant number of samples, statistics appears to overestimate the value of the potential biomarkers. Statistics is based on the assumption of an even distribution of the features across the training and test sets, that the findings can be generalized, and on the association with (in our example) gender only. This is apparently not even the case when using the data from 134 cases and controls. The expected result, that 95% of the significant biomarkers should stay significant in the test set, could not be observed. This may indicate that additional variables influence the outcome, and result in an overestimation of the statistical value. Especially when sample sizes are small, even statistically valid results must be interpreted with caution. In such situations, findings should be viewed as tentative and exploratory rather than conclusive. Our results further reveal that different machine learning algorithms perform similarly well, and seem to outperform linear classifiers. How-ever, we could also clearly demonstrate that the assessment of the performance of such a classifier can only be performed on an independent test set, the results obtained from the training set (even when performing leave-one-out cross validation) may be misleading. Based on the data presented here, it appears advisable to begin a study aiming at identification of biomarkers or classifiers by performing an analysis of 12 cases and controls, estimate sample size required for certain performance (e.g. accuracy of classification, level of confidence for biomarkers) based on re-sampling, and then perform the actual study with a sufficiently large set of samples. Potential biomarkers must pass WT, adjusted for multiple testing, preferably consistently when employing a set of > 30 resamples that each contain e.g. 70% of the available data. Classifiers are best established employing any of the available machine learning algorithms. The validity of both, biomarkers and classifiers, is generally overestimated in the training set, hence can only be addressed with confidence in an independent test set. The methods proposed here are independent on which clinical readout is considered. This fact has been shown by applying them to a dataset composed from diabetes type II either with normal kidney function or diabetic nephropathy. This last case study shows that the male-female case is reasonably representative of situations where the search for biomarkers and the classification tasks are rather involved.

## Methods

### Patients, Procedures and Demographics

Second morning urine samples were collected from apparently healthy volunteers in the course of the medical examination prior to employment at the Hannover Medical School. Consent was given by all participants. Samples were collected in 10 ml Sarstedt urine monovettes and frozen immediately after collection without the addition of any preservatives. All samples were collected anonymously, only age and gender were recorded. All samples were collected in Germany, and under German law this study does not require IRB approval.

### Sample preparation and CE-MS analysis

Urine samples were stored at 20°C for up to 3 years until analysis. For proteomic analysis, a 0.7 mL aliquot of urine was thawed immediately before use and diluted with 0.7 ml of 2 M urea, 10 mM NH4OH containing 0.02% SDS. To remove higher molecular mass proteins, samples were filtered using Centris-art ultracentrifugation filter devices (20 kDa molecular weight cut-off; Sartorius, Goettingen, Germany) at 3,000 rcf until 1.1 ml of filtrate was obtained. This filtrate was applied onto a PD-10 desalting column (Amersham Bioscience, Uppsala, Sweden) equilibrated in 0.01% NH4OH in HPLC-grade H2O (Roth, Germany) to remove urea, electrolytes, and salts. Finally, all samples were lyophilized, stored at 4°C, and suspended in HPLC-grade H2O shortly before CE-MS analysis, as described in [45]. CE-MS analysis was performed as described [45,46] using a P/ACE MDQ capillary electrophoresis system (Beckman Coulter, Fullerton, USA) on-line coupled to a Micro-TOF MS (Bruker Daltonic, Bremen, Germany). Data acquisition and MS acquisition methods were automatically controlled by the CE via contact-close-relays. The ESI spectra were accumulated every 3 s, over a range of m/z 350 to 3000 Th. Accumulation time has been chosen to be 3 s, since at peak width of ca. 15 sec at half peak height, essentially no resolution is lost when accumulating signal for 3 s. Faster sampling would result in any additional gain, but in loss in sensitivity, and also increase in the size of the data file. Accuracy, precision, selectivity, sensitivity, reproducibility, and stability are described in detail elsewhere [10,17,45]. In short, the detection limit is in the range of 1 fmol, depending on the ionization properties of the individual peptide. This corresponds to 100 - 1000 fmol/ml in a crude urine sample (before processing).

### Data processing

Mass spectral ion peaks representing identical molecules at different charge states were deconvoluted into single masses using MosaiquesVisu software [47]. Migration time and ion signal intensity (amplitude) were normalized based on 29 collagen fragments that serve as internal standards [17]. These internal polypeptide standards are the result of normal biological processes and have proven to be unaffected by any disease state studied to date (greater than 10,000 samples analysed to date) [48]. The resulting peak list characterizes each peptide by its molecular mass [Da], normalized migration time [min], and normalized signal intensity. All detected peptides were deposited, matched, and annotated in a Microsoft SQL database, allowing further analysis and comparison of multiple samples (patient groups). To establish the identity of peptides observed in different samples, a linear function was employed that allowed, depending on the mass of the polypeptide, a 50 ppm absolute mass deviation for peptides of 800 Da that increased linearly to 100 ppm absolute mass deviation for peptides with a maximum mass of 20 kDa. These values have been found appropriate in several recent studies [11,49,50], as a compromise between avoiding erroneous assignment of the same identity to two different peptides, and assigning two different identities to the same peptide in different analyses, due to mass deviation, especially at low abundance. A similar linear function was used when comparing CE migration times, allowing a 4% absolute deviation. CE-MS data of all individual samples can be accessed in Additional files 1, 2.

### Statistical methods, definition of biomarkers and sample classification

All the statistical analyses were implemented with internal scripts, using the R core software [51] as well as the contributed cran-packages ada, Kernlab, Ran-domForest, rpart, WilcoxCv, multtest, and ROCR available at http://cran.us.r-project.org.

## List of abbreviations used

1) AUC: area under the ROC curve; 2) AAC: area above the ROC curve; 3) BH: Benjamini-Hochberg; 4) CE-MS: capillary electrophoresis coupled mass spectrometry; 5) CD: diabetes type II with normal kidney function; 6) DN: diabetic nephropathy; 7) ESI: electro-spray ionization; 8) FDR: false discovery rate; 9) GLM: generalized linear model; 10) LC-MS: liquid chromatography coupled mass spectrometry; 11) LOD: limit of detection; 12) LOOCV: leave-one-out cross validation; 13) MALDI: matrix assisted laser desorption ionization; 14) MER: misclassification error rate; 15) *N*_diff_: differential sample size; 16) *N*_disc_: discriminative sample size; 17) ROC: receiver operating characteristic; 18) SQL: structured query language; 19) SVM: support vector machine; 20) WT: Wilcoxon rank sum test.

## Authors' information

Joost P Schanstra, Antonia Vlahou and Harald Mischak are all members of EUROKUP

## Authors' contributions

All authors participated in the design of the study. MD and HM performed the statistical analysis. HM performed the CE-MS analysis and initial data evaluation. AK, KH, SC, MD, and MG developed the high dimensional models. JPS and MH were involved in the recruitment of study participants. All authors were involved in drafting the manuscript, have read and approved the final manuscript.

## Supplementary Material

Additional file 1 **Training set data file for the male-female study**. The pivot table CE-MS-male-female-train.xls contains the amplitudes of each peptide in the 67 males (labelled as M...) and 67 females (labelled as F...) used for defining the biomarkers and training the classifiers for different *N*_train_. The pivot tables also contain a worksheet named "Peptides assignment" that shows the mass (Mass, in Da) and migration time (CE-Time, in min) of peptides assigned to a certain Pep:ID, which is subsequently utilized as the unique identifier in the database. Click here for file

Additional file 2 **Test set data file for the male-female study**. The table CE-MS-male-female-test.xls contains the amplitudes in the 67 males and 67 females used for testing the concordance of the biomarkers and estimating the errors of different classifiers. The pivot tables also contain a worksheet named "Peptides assignment" that shows the mass (Mass, in Da) and migration time (CE-Time, in min) of peptides assigned to a certain Pep:ID, which is subsequently utilized as the unique identifier in the database. Click here for file

Additional file 3 **Training set data file for the CD-DN study**. The pivot table CE-MS-DN-CN-train.xls contains the amplitudes of each peptide in the 60 DN (labelled as DN...) and 60 CD (labelled as CN...) used for defining the biomarkers and training the classifiers for different *N*_train_. Click here for file

Additional file 4 **Test set data file for the CD-DN study**. The table CE-MS-DN-CN-test.xls contains the amplitudes in the 60 DN and 60 CD used for testing the concordance of the biomarkers and estimating the errors of different classifiers. Click here for file

Additional file 5 **Typical effect size of a differentially expressed marker in CD versus DN case**. The distribution of two peptides, ID:67632 (upper panel) and ID:48751 (lower panel), was investigated in the complete training set (2 × 60) and 1000 re-sampled distributions. Typical effect size *δ *= (*μ*_CD _- *μ*_DN_)/*σ *(with *μ*_CD _and *μ*_DN _being the mean logarithmic intensity for a given peptide in the CD and DN populations, and σ the pooled standard deviation) is shown. Effect sizes as extreme as -4 and +8 are observed.
Click here for file

Additional file 6 **Concordance in the biomarkers using CD-DN data set**. The concordance of the markers defined using 60-CD and 60-DN subjects as training set in an independent test set of 60-CD and 60-DN is reported using 10 random splits of the total (2 × 120) data. On average, 447 markers are reported as being significant and 65% of them may be validated on average in the test data. Click here for file

Additional file 7 **Number of significant markers depends on sample size**. From the 2 × 60 training data, data sets of sizes *N*_diff _ranging from 7 to 60 were built via resampling. At each sample size the number of significant biomarkers (defined as having a *p*-value after BH adjustments < 0.05) is shown on the vertical axis. The procedure was repeated 10 times to generate the Box-Whisker plots. In all 10 experiments, no biomarkers could be declared significantly differentially expressed below a sample size of 7. Click here for file

Additional file 8 **Learning curve estimation of *N*_disc_**. Cohorts with sample sizes ranging from 2 × 7 - 2 × 58 (given on the x-axis) were arbitrarily generated out of the entire 2 × 60 CD-DN training dataset. 20 repetitions were performed for each size cohort. An SVM-based classifier was built for each dataset and its performance was tested in the independent test set. In the left hand panel the area above the curve AAC (AAC = 1-AUC) for each classifier is shown. The misclassification error rate MER is shown on the right. The red curves represent the mean AAC and mean MER. The inverse power law behaviour is obvious. Click here for file
