# Supplementary figures and images for: Addressing the Challenge of Defining Valid Proteomic Biomarkers and Classifiers
Source: BMC Bioinformatics. 2010 Dec 10;11:594. doi: 10.1186/1471-2105-11-594 (PMC3017845; doi:10.1186/1471-2105-11-594)

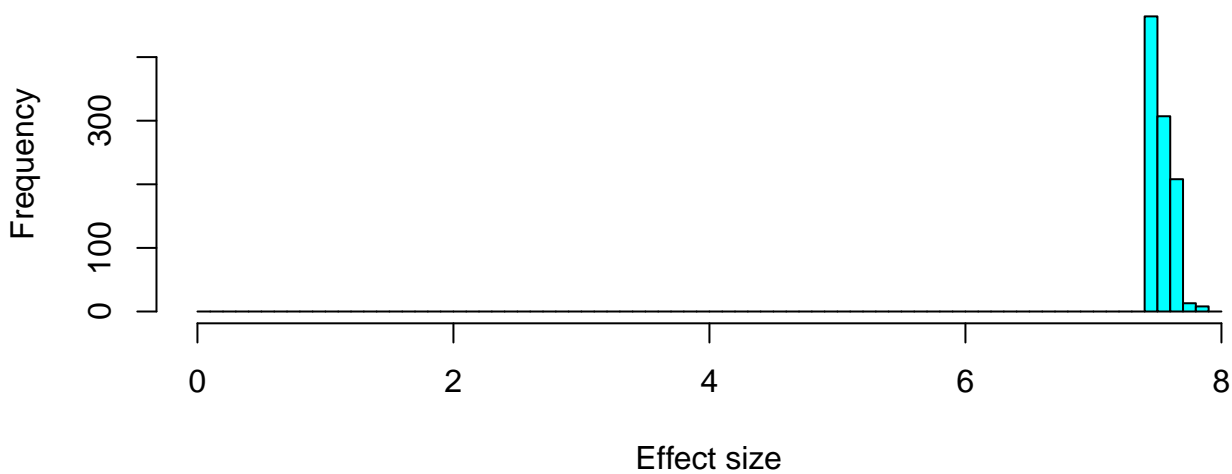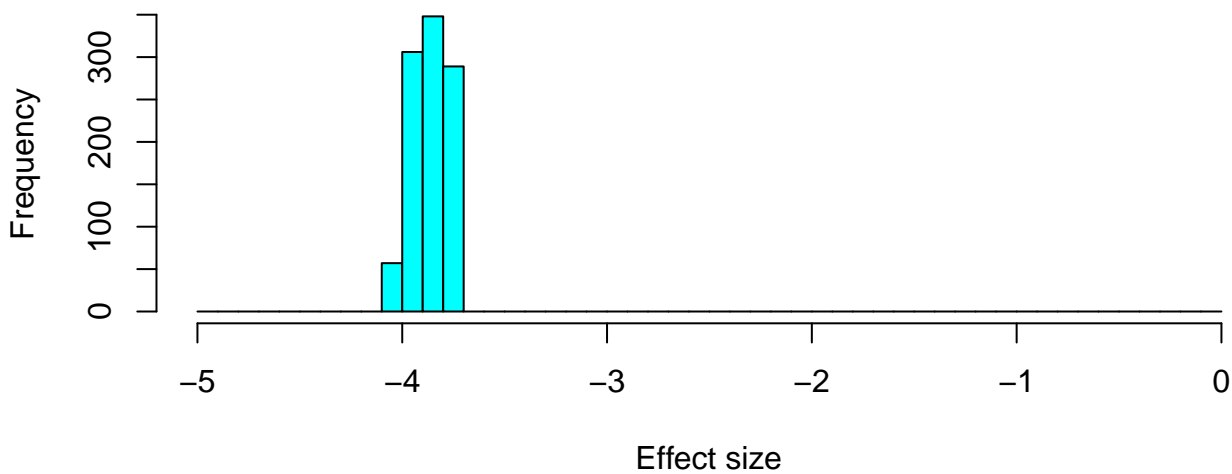

Supplement: Additional file 5 — Typical effect size of a differentially expressed marker in CD versus DN case. The distribution of two peptides, ID:67632 (upper panel) and ID:48751 (lower panel), was investigated in the complete training set (2 × 60) and 1000 re-sampled distributions. Typical effect size δ = (μCD - μDN)/σ (with μCD and μDN being the mean logarithmic intensity for a given peptide in the CD and DN populations, and σ the pooled standard deviation) is shown. Effect sizes as extreme as -4 and +8 are observed. [file 1471-2105-11-594-S5.PDF]
