# Supplementary material for: Addressing the Challenge of Defining Valid Proteomic Biomarkers and Classifiers
Source: BMC Bioinformatics. 2010 Dec 10;11:594. doi: 10.1186/1471-2105-11-594 (PMC3017845; doi:10.1186/1471-2105-11-594)
Supplement: Additional file 6 — Concordance in the biomarkers using CD-DN data set. The concordance of the markers defined using 60-CD and 60-DN subjects as training set in an independent test set of 60-CD and 60-DN is reported using 10 random splits of the total (2 × 120) data. On average, 447 markers are reported as being significant and 65% of them may be validated on average in the test data. [file 1471-2105-11-594-S6.PDF]

Supplementary Table 1:

Wilcox.train.ad	validated	% validated
418	280	67 %
464	307	66 %
391	288	73 %
434	309	71 %
400	263	65 %
464	299	64 %
471	294	63 %
525	313	60 %
491	291	60 %
405	259	64 %
